# Association Between Rotavirus Vaccination and Risk of Intussusception Among Neonates and Infants: A Systematic Review and Meta-analysis

**DOI:** 10.1001/jamanetworkopen.2019.12458

**Published:** 2019-10-04

**Authors:** Hai-Ling Lu, Ying Ding, Hemant Goyal, Hua-Guo Xu

**Affiliations:** 1 Department of Laboratory Medicine, Yancheng Traditional Chinese Medicine Hospital Affiliated to Nanjing University of Chinese Medicine, Yancheng, China; 2 Department of Pathology, The First Affiliated Hospital of Nanjing Medical University, Nanjing, China; 3 Department of Internal Medicine, Mercer University School of Medicine, Macon, Georgia; 4 Department of Laboratory Medicine, The First Affiliated Hospital of Nanjing Medical University, Nanjing, China

## Abstract

**Importance:**

The conclusions from the multiple randomized clinical trials exploring the relationship between development of intussusception and rotavirus vaccination among neonates and infants have been controversial.

**Objective:**

To evaluate the association between rotavirus vaccination and risk of intussusception.

**Data Sources:**

For this systematic review and meta-analysis, PubMed, Web of Science, Cochrane library, and Embase databases were searched from January 1, 1999, through December 31, 2018, using no language restrictions. The search terms were *rotavirus* or *RV* (rotavirus vaccine) or *HRV* (human rotavirus vaccine), vaccin*, and *intussusception*.

**Study Selection:**

Randomized clinical trials of neonates and infants that compared the risk of intussusception after the vaccination with a placebo group were included.

**Data Extraction and Synthesis:**

A fixed-effects model was used to pool the data. Statistical heterogeneity was assessed with Q test and *I*^2^ statistic; relative risk (RR), risk difference (RD), and 95% CIs were calculated using the Mantel-Haenszel method.

**Main Outcomes and Measures:**

The main outcome was the diagnosis of intussusception in the analysis. The pooled and subtotal results of RR, RD, and 95% CI for the risk of intussusception were estimated at 31 days, 1 year, and 2 years after vaccination.

**Results:**

A total of 25 randomized clinical trials including 200 594 participants (104 647 receiving vaccine and 95 947 receiving placebo) in 33 countries from 4 continents were included in this meta-analysis. Twenty cases of definite intussusception were diagnosed within 31 days after rotavirus vaccination, with 11 cases (55%) in the vaccine group and 9 cases (45%) in the placebo group (RD, 0.17 per 10 000 infants [95% CI, −1.16 to 1.50 per 10 000 infants], *P* = .80; RR, 1.14 [95% CI, 0.49 to 2.64], *P* = .77). Seventy-four cases were reported within 1 year, with 37 cases (50%) in the vaccine group and 37 cases (50%) in the placebo group (RD, −0.65 per 10 000 infants [95% CI, −2.68 to 1.39 per 10 000 infants], *P* = .53; RR, 0.84 [95% CI, 0.53 to 1.32], *P* = .45). Fifty-nine cases were reported within 2 years, with 29 cases (49%) in the vaccine group and 30 cases (51%) in the placebo group (RD, −0.48 per 10 000 infants [95% CI, −3.64 to 2.69 per 10 000 infants], *P* = .77; RR, 0.91 [95% CI, 0.55 to 1.52], *P* = .73).

**Conclusions and Relevance:**

Results of this systematic review and meta-analysis suggest that monovalent, pentavalent, monovalent human-bovine, oral bovine pentavalent, and human neonatal rotavirus vaccination was not associated with an elevated risk of intussusception among neonates or infants.

## Key Points

**Question:**

What is the association between rotavirus vaccination and risk of intussusception?

**Findings:**

In this systematic review and meta-analysis of 25 randomized clinical trials including 200 594 participants (104 647 receiving vaccine and 95 947 receiving placebo) in 33 countries from 4 continents, monovalent, pentavalent, monovalent human-bovine, oral bovine pentavalent, and human neonatal rotavirus vaccinations were not associated with an increased risk of intussusception compared with placebo for up to 2 years after vaccination.

**Meaning:**

The findings suggest that rotavirus vaccination is not associated with an increased risk of intussusception for up to 2 years after vaccination among neonates or infants.

## Teaser

This systematic review and meta-analysis assesses the association of rotavirus vaccination among neonates and infants with risk of intussusception for up to 2 years after vaccination overall and by type of vaccine.

## Introduction

Vaccines play an important role in the prevention of infectious diseases. Rotavirus (RV) vaccination has significantly reduced the occurrence and severity of RV-related gastroenteritis and mortality among infants and young children.^1^ Although data from some clinical trials show that the efficacy of RV vaccines for prevention of RV gastroenteritis reaches 36% to 96% within a year of follow-up,^2,3^ the Global Advisory Committee on Vaccine Safety has noted that the use of RV vaccines may be associated with an increased risk of intussusception.^4^ Even though the efficacy of the vaccine might outweigh the small potential risk of intussusception, the Global Advisory Committee on Vaccine Safety has also suggested performance of active surveillance to ensure that the long-term benefit and safety of RV vaccines are entirely assessed.^4^

Data from randomized clinical trials (RCTs) regarding the efficacy and safety of RV vaccines show conflicting evidence on the incidence of intussusception. Whether or not there was an association between vaccination and an increased risk of intussusception, the answer varied across studies.^5,6,7,8^ The varying data from the RCTs about the difference in the incidence of intussusception could be attributable to multiple variables such as age, sex, geographic and population distribution of the participants, and the different types of RV vaccines used in these studies. However, the reasons behind the differing risk of intussusception are still not clear. Therefore, we conducted this systematic review and meta-analysis of published RCTs to further assess the risk of development of intussusception after RV vaccination.

## Methods

This systematic review and meta-analysis followed the Cochrane Collaboration Group^9^ and Preferred Reporting Items for Systematic Reviews and Meta-analyses (PRISMA)^10^ reporting guidelines. The construction of databases, article screening, article quality evaluation, and data extraction were independently completed by 2 of us (H.-L.L. and Y.D.). Discrepancies were resolved by consensus or, if necessary, with the assistance of one of us (H.-G.X.).

### Literature Search and Study Selection

The PubMed, Web of Science, Cochrane library, and Embase databases were searched from January 1, 1999, through December 31, 2018, with no language restrictions. The search teams were ([*rotavirus* or *RV* (rotavirus) or *HRV* (human rotavirus vaccine)] and vaccin*) and (*intussusception* or *intestinal invagination* or *indignation* or *invagination intextinorum*) and (*Clinical Trial[PTyp] and (1999/01/01[PDat]:2018/12/31[PDat]*). In addition, references from the relevant articles were searched for appropriate studies.

**Figure 1.**
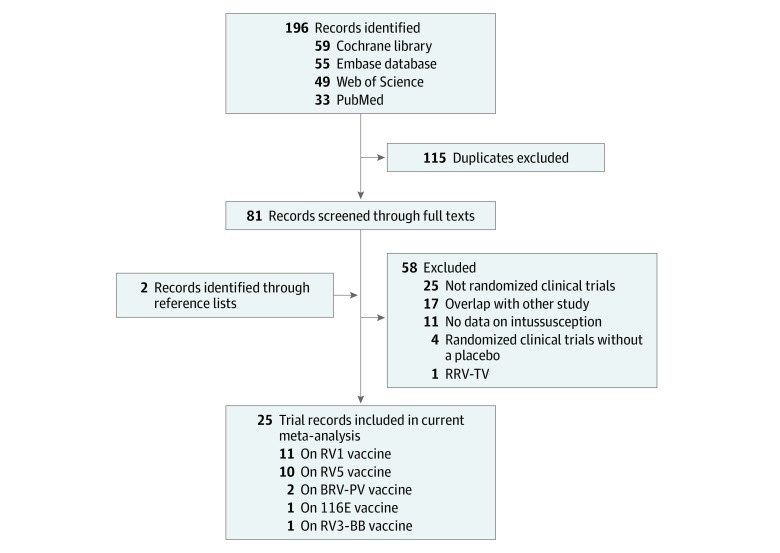
Flow Diagram BRV-PV indicates oral bovine rotavirus pentavalent vaccine (Rotasiil); RRV-TV, human reassortant rotavirus tetravalent vaccine; RV1, monovalent rotavirus vaccine (Rotarix); RV3-BB, human neonatal rotavirus vaccine; RV5, pentavalent rotavirus vaccine (Rotateq); 116E, monovalent human-bovine rotavirus vaccine (Rotavac).

This meta-analysis focused on the association between RV vaccination and the risk of intussusception. Only RCTs that compared the risk of intussusception between the vaccine and placebo groups for neonates or infants were included in the analysis. Other inclusion criteria included the following: (1) use of any type of vaccination, such as monovalent (RV1) (Rotarix; GlaxoSmithKline), pentavalent (RV5) (RotaTeq; Merck & Co, Inc), monovalent human-bovine (116E) (Rotavac; Bharat Biotech), oral bovine pentavalent (BRV-PV) (Rotasiil; Serum Institute of India), and human neonatal (RV3-BB); (2) a sample size of at least 100 participants; and (3) data on the incidence of intussusception. Exclusion criteria were (1) no data on intussusception; (2) no placebo group; (3) use of human reassortant rotavirus tetravalent vaccine (RRV-TV) (Rotashield; Wyeth Laboratories Inc) because the US Advisory Committee on Immunization no longer recommends the use of this vaccine owing to high risk of intussusception; and (4) duplicate publications. Some studies included the participants from the same general population, in which case, the most comprehensive and up-to-date study was selected for inclusion in this meta-analysis. Data from unpublished trials or conference abstracts were also excluded from the final analysis (Figure 1).

### Data Collection and Extraction

Data were extracted using a standardized data extraction form. Information collected included the study title, authors, publication year, study period, phase and the registration number of the RCT, city, country, continent, sex, weight, vaccine type, time of vaccination, sample size in the vaccine or placebo group, number of intussusception cases, number of days between the administration of the vaccine and diagnosis of intussusception, the risk estimates or data used to calculate the risk estimates, and 95% CIs or data used to calculate 95% CIs. If a trial had more than 2 groups or differed in the vaccine component or concentration, the extracted information and data on the vaccine used were those of the similar composition and with the closest concentration to the approved vaccine.

### Statistical Analysis

The association between RV vaccination and intussusception, the pooled results of relative risk (RR), the risk difference (RD), and 95% CIs in the 3 different follow-up periods were calculated using the Mantel-Haenszel method. Because of the very low incidence of intussusception, the number of intussusceptions in the vaccine and placebo groups during the observation period was 0 at the same time in some trials. To accurately and objectively reflect the facts, we evaluated RR and RD. Undefined RRs (ie, RR of 0 or infinity) were not included in the statistical calculations but were included in the calculation of RD.

Separate analyses were performed for RV1 and RV5. Because the 116E, BRV-PV, and RV3-BB vaccines were used only in local areas and there were fewer related trials, these 3 vaccines were combined into 1 group for analysis. If only 1 trial evaluated the vaccine, a systematic review was conducted, and if more than 1 trial evaluated the vaccine, a meta-analysis was conducted. The results were separately calculated as subtotal results. The pooled and subtotal results were presented in forest plots as RRs, RDs, and 95% CIs of intussusception after RV vaccination. Both the pooled and subtotal results of RR and RD of intussusception were estimated at 31 days, 1 year, and 2 years after the vaccination period. If the number of trials was small during a follow-up period (31 days, 1 year, and 2 years), analysis was not conducted on publication bias.

The Q test and *I*^2^ statistic index were used to assess the degree of heterogeneity between different studies: low (<25%), moderate (26%-50%), and high (51%-75%). Results were calculated using a fixed- or random-effect model depending on the statistical results of the heterogeneity test. Sensitivity analysis was performed for identifying the heterogeneity among the studies. All statistical analyses were performed using Stata, version 14.2 (StataCorp). A 2-sided *P* < .05 was considered to indicate statistical significance.

## Results

### Study Screening

A total of 25 RCTs met the inclusion criteria. The initial search produced 196 studies from all 4 databases. There were 33 studies from PubMed, 49 studies from Web of Science, 59 studies from Cochrane library, and 55 studies from Embase. After the screening of titles and abstracts, 115 studies were removed because of duplicates. The full texts of the remaining 81 studies were reviewed in detail. The reference lists or relevant publications of these articles were also screened based on the eligibility criteria; 58 studies were excluded, and 2 studies were newly identified through the references. The selection process is summarized in Figure 1.

### Study Characteristics

As shown in Table 1, a total of 25 RCTs including 200 594 participants (104 647 receiving vaccine and 95 947 receiving placebo) in 33 countries from 4 continents were finally included in this systematic review and meta-analysis. There were 11 trials on the RV1 vaccine, 10 trials on the RV5 vaccine, 2 trials on the BRV-PV vaccine, and 1 trial on the 116E and RV3-BB vaccines. Most RCTs reported intussusception cases from up to 31 days after vaccination. As shown in Table 2, 20 cases of definite intussusception were diagnosed within 31 days after RV vaccination, with 11 cases (55%) in the vaccine group and 9 cases (45%) in the placebo group. A total of 74 cases of intussusception (37 cases [50%] in the vaccine group and 37 cases [50%] in the placebo group) were reported within 1 year and 59 cases (29 cases [49%] in the vaccine and 30 cases [51%] in the placebo group) within 2 years after vaccination.

**Table 1.** Characteristics of the Included Randomized Clinical Trials

Source	Countries or Regions	Vaccine	Study Period	Clinical Trial Phase	Registration No.	Queue, No.	Age at First Dose	Participants, No.	
								Vaccine Group	Placebo Group
Dennehy et al,^11^ 2005	United States and Canada	RV1	December 2000-September 2001	2	NA	2	5-15 wk	209	108
Kawamura et al,^12^ 2011	Japan	RV1	June 2007-December 2009	3	NCT00480324	1	7.7 (2.01) wk^a^	507	257
Li et al,^13^ 2014	China	RV1	August 2010-December 2010	3	NCT01171963	1	NA	1666	1667
Madhi et al,^14^ 2010	South Africa and Malawi	RV1	2005-2007	NA	NCT00241644	1	NA	3298	1641
Phua et al,^15^ 2009	China and Singapore	RV1	December 2003-August 2005	3	NCT00329745	1	NA	5359	5349
Ruiz-Palacios et al,^16^ 2006	Latin America (11 countries) and Finland	RV1	August 2003-March 2004	3	NCT00139347 and NCT00263666	1	2-4 mo	31 673	31 552
Salinas et al,^17^ 2005	Latin America (3 countries)	RV1	May 2001-April 2003	NA	NA	3	8.3 wk^a^	540	537
Steele et al,^18^ 2010	South Africa	RV1	September 2003-October 2004	2	NCT00383903, eTrack 444563/013	2	5-10 wk	190	96
Tregnaghi et al,^19^ 2011	Latin America (6 countries)	RV1	December 2003-March 2007	3	NCT00139347	1	NA	4376	2192
Vesikari et al,^20^ 2004	Finland	RV1	August 2000-November 2000	NA	NA	1	6-12 wk	270	135
Vesikari et al,^3^ 2007	Europe (6 countries)	RV1	September 2004-February 2005	3b	NCT00140686, eTrack102247	1	6-14 wk	2646	1348
Armah et al,^21^ 2010	Ghana, Kenya, and Mali	RV5	April 2007-May 2009	NA	NCT00362648	1	NA	2733	2735
Chang et al,^22^ 2009	China	RV5	April 2003-June 2004	3	NA	1	6-12 wk	95	93
Grant et al,^23^ 2012	United States	RV5	March 2002-October 2003	NA	NA	1	NA	512	494
Iwata et al,^24^ 2013	Japan	RV5	August 2008-August 2009	NA	NCT00718237	1	6-12 wk	380	381
Kim et al,^25^ 2008	South Korea	RV5	August 2005-July 2006	3	NA	1	9 wk^b^	115	63
Mo et al,^26^ 2017	China	RV5	May 2014-October 2014	NA	NCT02062385	1	6-12 wk	2015	2019
Rodriguez et al,^27^ 2007	11 Countries	RV5	2001-2005	NA	NA	1	6-12 wk	662	696
Vesikari et al,^28^ 2006	Finland	RV5	1998-2001	2	NA	3	2-8 mo	323	322
Vesikari et al,^29^ 2006	11 Countries, including United States and Finland	RV5	2001-2004	3	NCT00090233	1	9.8 (1.4) wk^a^	34 644	34 630
Zaman et al,^30^ 2010	Bangladesh and Vietnam	RV5	March 2007-March 2009, September 2007-March 2009	NA	NCT00362648	1	8.9 (1.5) wk^a^	1018	1018
Bhandari et al,^31^ 2014	India	116E	March 2011-November 2012	NA	NCT01305109	1	6.8 wk^a^	4532	2267
Isanaka et al,^32^ 2017	Niger	BRV-PV	August 2014-November 2015	3	NCT02145000	1	6-8 wk	2044	2047
Kulkarni et al,^2^ 2017	India	BRV-PV	2014-2016	3	NCT02133690	1	48.2 (4.1) d^a^	3749	3751
Bines et al,^33^ 2018	Indonesia	RV3-BB	January 2013-July 2016	NA	ACTRN12612001282875	1	0-5 d and 8-10 wk	1091	549

Abbreviations: BRV-PV, oral bovine rotavirus pentavalent vaccine (Rotasiil); NA, not applicable; RV1, monovalent rotavirus vaccine (Rotarix); RV3-BB, human neonatal rotavirus vaccine; RV5, pentavalent rotavirus vaccine (Rotateq); 116E, monovalent human-bovine rotavirus vaccine (Rotavac).

^a^ Data are presented as mean or mean (SD).

^b^ Data are presented as median.

**Table 2.** Meta-analysis Results of the Risk of Intussusception After Rotavirus Vaccination

Vaccine Type, Source	Intussusception 31 d After Each Dose^a^				Intussusception at 1 y				Intussusception at 2 y			
	No. of Cases		RR (95% CI)	RD (95% CI), per 10 000 Infants	No. of Cases		RR (95% CI)	RD (95% CI), per 10 000 Infants	No. of Cases		RR (95% CI)	RD (95% CI), per 10 000 Infants
	Vaccine Group	Placebo Group			Vaccine Group	Placebo Group			Vaccine Group	Placebo Group		
RV1												
Dennehy et al,^11^ 2005^b^	0	0	NA	0 (−142.96 to 142.96)	0	0	NA	0 (−142.96 to 142.96)	NA	NA	NA	NA
Kawamura et al,^12^ 2011	0	0	NA	0 (−60.20 to 60.20)	0	0	NA	0 (−60.20 to 60.20)	NA	NA	NA	NA
Li et al,^13^ 2014	0	0	NA	0 (−11.75 to 11.75)	1	1	1.00 (0.06 to 15.98)	0 (−16.61 to 16.62)	NA	NA	NA	NA
Madhi et al,^14^ 2010	0	0	NA	0 (−9.43 to 9.43)	1	0	1.49 (0.06 to 36.63)	3.03 (−8.11 to 14.17)	NA	NA	NA	NA
Phua et al,^15^ 2009	0	0	NA	0 (−3.66 to 3.66)	NA	NA	NA	NA	8	4	2.00 (0.60 to 6.63)	7.45 (−5.22 to 20.12)
Ruiz-Palacios et al,^16^ 2006	6	7	0.85 (0.29 to 2.54)	−0.32 (−2.56 to 1.91)	9	16	0.56 (0.25 to 1.27)	−2.23 (−5.33 to 0.87)	NA	NA	NA	NA
Salinas et al,^17^ 2005^b^	0	0	NA	0 (−36.31 to 36.31)	NA	NA	NA	NA	NA	NA	NA	NA
Steele et al,^18^ 2010	0	0	NA	0 (−159.87 to 159.87)	NA	NA	NA	NA	NA	NA	NA	NA
Tregnaghi et al,^19^ 2011	NA	NA	NA	NA	4	2	1.00 (0.18 to 5.47)	0.02 (−15.47 to 15.51)	NA	NA	NA	NA
Vesikari et al,^20^ 2004	0	0	NA	0 (−113.83 to 113.83)	0	0	NA	0 (−113.83 to 113.83)	0	0	NA	0 (−113.83 to 113.83)
Vesikari et al,^3^ 2007	1	0	1.53 (0.06 to 37.51)	3.78 (−9.92 to 17.48)	1	0	1.53 (0.06 to 37.51)	3.78 (−9.92 to 17.48)	2	1	1.02 (0.09 to 11.23)	0.14 (−17.77 to 18.05)
RV5												
Armah et al,^21^ 2010	0	0	NA	0 (−7.17 to 7.17)	0	0	NA	0 (−7.17 to 7.17)	NA	NA	NA	NA
Chang et al,^22^ 2009	0	0	NA	0 (−205.80 to 205.80)	0	0	NA	0 (−205.80 to 205.80)	NA	NA	NA	NA
Grant et al,^23^ 2012	0	0	NA	0 (−38.89 to 38.89)	0	0	NA	0 (−38.89 to 38.89)	NA	NA	NA	NA
Iwata et al,^24^ 2013	0	0	NA	0 (−51.34 to 51.34)	NA	NA	NA	NA	NA	NA	NA	NA
Kim et al,^25^ 2008	0	0	NA	0 (−246.45 to 246.45)	NA	NA	NA	NA	NA	NA	NA	NA
Mo et al,^26^ 2017	0	0	NA	0 (−9.71 to 9.71)	2	0	5.01 (0.24 to 104.29)	9.93 (−6.90 to 26.75)	NA	NA	NA	NA
Rodriguez et al,^27^ 2007	1	0	3.15 (0.13 to 77.28)	15.11 (−26.16 to 56.37)	NA	NA	NA	NA	NA	NA	NA	NA
Vesikari et al,^28^ 2006^b^	0	0	NA	0 (−60.54 to 60.54)	0	0	NA	0 (−60.54 to 60.54)	0	0	NA	0 (−60.54 to 60.54)
Vesikari et al,^29^ 2006	3	2	1.50 (0.25 to 8.97)	0.29 (−0.98 to 1.55)	12	15	0.80 (0.37 to 1.71)	−0.87 (−3.81 to 2.07)	12	18	0.67 (0.32 to 1.38)	−1.73 (−4.83 to 1.36)
Zaman et al,^30^ 2010	0	0	NA	0 (−19.23 to 19.23)	0	1	0.33 (0.01 to 8.17)	−9.82 (−37.01 to 17.36)	NA	NA	NA	NA
116E, Rotavac												
Bhandari et al,^31^ 2014	0	0	NA	0 (−6.83 to 6.83)	6	2	1.50 (0.30 to 7.43)	4.42 (−11.75 to 20.59)	NA	NA	NA	NA
BRV-PV, Rotasiil												
Isanaka et al,^32^ 2017	0	0	NA	0 (−9.58 to 9.58)	0	0	NA	0 (−9.58 to 9.58)	0	0	NA	0 (−9.58 to 9.58)
Kulkarni et al,^2^ 2017	0	0	NA	0 (−5.22 to 5.22)	NA	NA	NA	NA	6	7	0.86 (0.29 to 2.55)	−2.66 (−21.49 to 16.17)
RV3-BB												
Bines et al,^33^ 2018	0	0		0 (−28.20 to 28.20)	1	0	1.51 (0.06 to 37.03)	9.17 (−24.25 to 42.59)	1	0	1.51 (0.06 to 37.03)	9.17 (−24.25 to 42.59)
Fixed-effects model using Mantel-Haenszel	11	9	1.14 (0.49 to 2.64)	0.17 (−1.16 to 1.50)	37	37	0.84 (0.53 to 1.32)	−0.65 (−2.68 to 1.39)	29	30	0.91 (0.55 to 1.52)	−0.48 (−3.64 to 2.69)
*P* value	NA	NA	.77	.80	NA	NA	.45	.53	NA	NA	.73	.77

Abbreviations: BRV-PV, oral bovine rotavirus pentavalent vaccine (Rotasiil); NA, not applicable; RD, risk difference; RR, relative risk; RV1, monovalent rotavirus vaccine (Rotarix); RV3-BB, human neonatal rotavirus vaccine; RV5, pentavalent rotavirus vaccine (Rotateq); 116E, monovalent human-bovine rotavirus vaccine (Rotavac).

^a^ From the data extracted for the study, most of the intussusception data description was divided by 31 days; thus, 31 days was chosen as the statistical indicator. We believe that the 31-day follow-up reflected the short-term effect of the vaccine; the long-term effect was shown at 1 and 2 years.

^b^ The 3 studies compared different concentrations of vaccine vs placebo. Among 4630 patients, 2 cases of intussusception occurred in the low-concentration group; 1 case was a 7-month-old boy, with occurrence 9 days after the first dose of RV5, and the second case was a 10-month-old boy, with occurrence 6 months after the second dose of RV1.

### Study Quality

Quality assessment of the trials was performed according to the Cochrane collaboration′s tool for assessing the risk of bias.^9^ Of the 25 RCTs, 19 were high quality and 6 were moderate quality.

### Meta-analysis

#### Risk of Intussusception Within 31 Days After Rotavirus Vaccination

**Figure 2.**
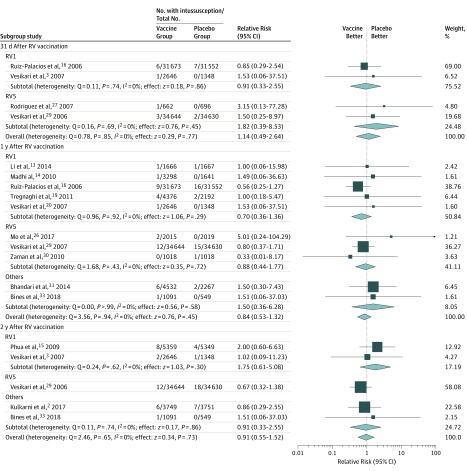
Subgroup Analysis for Intussusception Between Rotavirus (RV) Vaccine and Placebo Groups at Different Follow-up Times Relative risk and 95% CIs were calculated using the Mantel-Haenszel method, with a fixed-effects model used to pool data. Randomized clinical trials with 0 cases of intussusception among the vaccine and placebo groups were not included in the relative risk statistics but were included in the statistics of the risk difference. Other vaccines included monovalent human-bovine (116E) (Rotavac), human neonatal (RV3-BB), and oral bovine pentavalent (BRV-PV). Boxes represent means, with the size of the box corresponding with the weight; horizontal lines represent 95% CIs; and diamonds indicate pooled means with the horizontal points of the diamonds representing 95% CIs. RV1 indicates monovalent rotavirus vaccine (Rotarix); RV5, pentavalent rotavirus vaccine (Rotateq).

As shown in Table 2 and Figure 2, 20 cases of intussusception were diagnosed within 31 days after any RV vaccination, with 11 cases (55%) in the vaccine group and 9 cases (45%) in the placebo group. The RR of intussusception ranged from 0.85 to 3.15 among the 4 studies that reported intussusception risk within 31 days after vaccination. Heterogeneity among these studies was low (Q = 0.78; *P* = .85; *I*^2^ = 0%). The RD of intussusception ranged from −0.32 per 10 000 infants to 15.11 per 10 000 infants (Table 2). Heterogeneity among those studies was also very low (Q = 1.01; *P* > .99; *I*^2^ = 0%). The pooled effects were calculated using the fixed-effect model. The overall estimate of RR for intussusception within 31 days for the fixed-effect model was 1.14 (95% CI, 0.49-2.64; *P* = .77). The overall estimate of RD of intussusception within 31 days after each dose for the fixed-effect model was 0.17 per 10 000 infants (95% CI, −1.16 to 1.50 per 10 000 infants; *P* = .80).

Fourteen cases of intussusception were diagnosed within 31 days after RV1 vaccination (7 cases [50%] in the vaccine group and 7 cases [50%] in the placebo group). The subtotal estimate of RR of intussusception within 31 days after each dose of RV1 for the fixed-effect model was 0.91 (95% CI, 0.33-2.55; *P* = .86). The subtotal estimate of RD of intussusception within 31 days after each dose for the fixed-effect model was −0.08 per 10 000 infants (95% CI, −2.22 to 2.06 per 10 000 infants; *P* = .94).

Six cases of intussusception were diagnosed within 31 days after RV5 vaccination (4 cases [66%] in the vaccine group and 2 cases [33%] in the placebo group). The subtotal estimate of RR of intussusception within 31 days after each RV5 dose for the fixed-effect model was 1.82 (95% CI, 0.39-8.53; *P* = .45). The subtotal estimate of RD of intussusception in 31 days after each RV5 dose for the fixed-effect model was 0.48 per 10 000 infants (95% CI, −1.32 to 2.27 per 10 000 infants; *P* = .60).

#### Risk of Intussusception Within 1 Year of Vaccination

As shown in Table 2 and Figure 2, a total of 74 cases of definite intussusception were diagnosed within 1 year after any RV vaccination (37 cases [50%] in each group). The RR of intussusception ranged from 0.33 to 5.01 among 10 studies that reported intussusception outcome at 1 year. Heterogeneity among these studies was low (Q = 3.56; *P* = .94; *I*^2^ = 0%). The RD of intussusception ranged from −9.82 to 9.93 per 10 000 infants, with low heterogeneity (Q = 4.57; *P* > .99; *I*^2^ = 0%). The pooled effects were calculated using the fixed-effect model. The overall estimate of RR of intussusception within 1 year of RV vaccination for the fixed-effect model was 0.84 (95% CI, 0.53-1.32; *P* = .45). The overall estimate of RD of intussusception within 1 year after each RV dose for the fixed-effect model was –0.65 per 10 000 infants (95% CI, −2.68 to 1.39 per 10 000 infants; *P* = .53).

Thirty-five cases of definite intussusception were diagnosed within 1 year after RV1 vaccination (16 cases [46%] in the vaccine group and 19 cases [54%] in the placebo group). The subtotal estimate of RR of intussusception within 1 year after each dose of RV1 for the fixed-effect model was 0.70 (95% CI, 0.36-1.36; *P* = .29). The subtotal estimate of RD of intussusception within 1 year after receipt of each dose for the fixed-effect model was −1.40 per 10 000 infants (95% CI, −4.38 to 1.59 per 10 000 infants; *P* = .36).

Thirty cases of intussusception were identified within 1 year after RV5 vaccination (14 cases [47%] in the vaccine group and 16 cases [53%] in the placebo group). The subtotal estimate of RR of intussusception within 1 year after each dose for the fixed-effect model was 0.88 (95% CI, 0.44-1.77; *P* = .72). The subtotal estimate of RD of intussusception within 1 year after each dose for the fixed-effect model was −0.48 per 10 000 infants (95% CI, −3.33 to 2.36 per 10 000 infants; *P* = .74).

Nine cases of intussusception were diagnosed within 1 year after 116E and RV3-BB vaccinations (7 cases [78%] in the vaccine group and 2 cases [22%] in the placebo group). The subtotal estimate of RR of intussusception within 1 year after each dose of these vaccines for the fixed-effect model was 1.50 (95% CI, 0.36-6.28; *P* = .58). The subtotal estimate of RD of intussusception within 1 year after each dose for the fixed-effect model was 3.46 per 10 000 infants (95% CI, −6.55 to 13.47 per 10 000 infants; *P* = .50).

#### Risk of Intussusception Within 2 Years of Vaccination

As shown in Table 2 and Figure 2, a total of 59 cases of intussusception were diagnosed in the 5 studies that reported outcome within 2 years after any RV vaccination (29 cases [49%] in the vaccine group and 30 cases [51%] in the placebo group). The RR of intussusception ranged from a minimum of 0.67 to a maximum of 2.00 with low heterogeneity among these studies (Q = 2.46; *P* = .65; *I*^2^ = 0%). The RD of intussusception ranged from −2.66 to 9.17 per 10 000 infants. Heterogeneity among those studies was also low (Q = 2.52; *P* = .93; *I*^2^ = 0%). The pooled effects were calculated using the fixed-effect model. The overall estimate of RR of intussusception within 2 years after vaccination for the fixed-effect model was 0.91 (95% CI, 0.55-1.52; *P* = .73). The overall estimate of RD of intussusception within 2 years after each dose for the fixed-effect model was −0.48 per 10 000 infants (95% CI, −3.64 to 2.69 per 10 000 infants; *P* = .77).

Fifteen cases of definite intussusception were diagnosed within 2 years of RV1 vaccination (10 cases [67%] in the vaccine group and 5 cases [33%] in the placebo group). The subtotal estimate of RR of intussusception within 2 years after each dose for the fixed-effect model was 1.75 (95% CI, 0.61-5.08; *P* = .30). The subtotal estimate of RD of intussusception within 2 years after each dose for the fixed-effect model was 5.48 per 10 000 infants (95% CI, –5.14 to 16.11 per 10 000 infants; *P* = .31).

Thirty cases of definite intussusception were diagnosed within 2 years of RV5 vaccination (12 cases [40%] in the vaccine group and 18 cases [60%] in the placebo group). The subtotal estimate of RD of intussusception within 2 years after each dose of RV5 for the fixed-effect model was −1.72 per 10 000 infants (95% CI, −4.84 to 1.40 per 10 000 infants; *P* = .28).

Fourteen cases of definite intussusception were diagnosed within 2 years after BRV-PV and RV3-BB vaccinations (7 cases [50%] in the vaccine group and 7 cases [50%] in the placebo group). The subtotal estimate of RR of intussusception within 2 years after each dose of BRV-PV and RV3-BB vaccines for the fixed-effect model was 0.91 (95% CI, 0.33-2.55; *P* = .86). The subtotal estimate of RD of intussusception within 2 years after each dose for the fixed-effect model was −0.50 per 10 000 infants (95% CI, −12.34 to 11.34 per 10 000 infants; *P* = .93).

## Discussion

Intussusception is a potentially life-threatening condition in children, and recent evidence has indicated an association between the RV vaccination and intussusception.^5,34^ Because of this adverse event, careful monitoring for development of intussusception after the administration of RV vaccine is suggested. In this systematic review and meta-analysis of RCTs evaluating the risk of intussusception after RV vaccination found no such significant association. This meta-analysis included the RCTs that used RV1, RV5, 116E, BRV-PV, or RV3-BB vaccine. Analysis of the subtotal group of different vaccine types and the pooled estimated risks of intussusception within 31 days after each dose, and 1 and 2 years after vaccination revealed no association of risk of developing intussusception after receipt of the rotavirus vaccine, a finding that corresponds with the results of some previous studies.^6,7,35,36,37^

The absence of any significant association between the RV vaccine and intussusception could possibly be attributed to a wide range of RCTs covering a total of 200 594 infants worldwide. The key strength of this meta-analysis was the large number of infants included in the RCTs, which focused on surveillance of vaccine safety. Of the total 108 cases of intussusception, 3 occurred within 7 days^16,29^ and after the second dose of the vaccine. However, there was no statistical difference in the incidence of intussusception between the vaccine group and the placebo group. Studies^5,34,38,39,40^ with different methods, such as cohort studies, case-control studies, self-controlled case series (SCCS), or self-controlled risk interval evaluation studies, reported a positive association between RV vaccination and intussusception, whereas RCTs often found no correlation between intussusception and vaccination. A recent meta-analysis^41^ of 6 cohort studies (4 506 265 first doses) and 5 case-control studies (n = 9643 infants) suggests that the RV vaccination is associated with an increased risk of development of intussusception, which was predominantly seen after the administration of the first dose. Another meta-analysis conducted by Dong et al^42^ that included children receiving RV1 and RV5 vaccines showed an increased risk of intussusception within 7 days, especially after the first dose. However, only SCCS and self-controlled risk interval studies were included in the analysis. Another meta-analysis of 10 SCCSs showed that RR for intussusception was 5.71 (95% CI, 4.50-7.25) from 1 to 7 days after the first dose, 1.69 (95% CI, 1.33-2.14) after the second dose, and 1.14 (95% CI, 0.75-1.74) after the third dose.^43^ The SCCS evaluation is increasingly being used during the active vaccine safety surveillance, whereas an SCCS has its own limitations of measuring only the incidence of reported cases with a descriptive design rather than an analytic study. Thus, SCCSs could include potential referral bias because variation of the treatment application has no control.^44^ When comparing different study designs to determine the best design for surveillance of vaccine safety, the limitations of those studies are evident, especially based on their heterogeneity. Thus, these positive results need to be carefully considered and further investigated.

Another possible reason for no association could be the exclusion of RRV-TV vaccination in this meta-analysis. Many previous studies^45,46,47^ investigating RRV-TV demonstrated that RRV-TV was associated with a strong increased risk of intussusception; it was suspended in 1999 because of the safety issues.

### Limitations

This study has limitations. The power was low for analysis of RCTs assessing the risk of intussusception with 116E, BRV-PV, and RV3-BB vaccines because of the limited number of trials. Another limitation was the inability to assess whether there was a difference in the risk of intussusception among infants from various geographic regions because of unavailability of sufficiently large trials in the same region.

## Conclusions

In this systematic review and meta-analysis of RCTs of the RV1, RV5, 116E, BRV-PV, and RV3-BB vaccines, we found no association of vaccination with increased risk of intussusception compared with placebo among infants for up to 2 years after vaccination. Our results contradict the postmarketing monitoring suggestion about the risk of intussusception after the RV vaccination. We suggest that the benefit of the vaccination exceeds the potential risk of intussusception.
